# Vanadium disulfide flakes with nanolayered titanium disulfide coating as cathode materials in lithium-ion batteries

**DOI:** 10.1038/s41467-019-09400-w

**Published:** 2019-04-16

**Authors:** Lu Li, Zhaodong Li, Anthony Yoshimura, Congli Sun, Tianmeng Wang, Yanwen Chen, Zhizhong Chen, Aaron Littlejohn, Yu Xiang, Prateek Hundekar, Stephen F. Bartolucci, Jian Shi, Su-Fei Shi, Vincent Meunier, Gwo-Ching Wang, Nikhil Koratkar

**Affiliations:** 1 Department of Mechanical, Aerospace and Nuclear Engineering, Rensselaer Polytechnic Institute, 110 8th Street, Troy, NY 12180 USA; 2 Department of Physics, Applied Physics, and Astronomy, Rensselaer Polytechnic Institute, 110 8th Street, Troy, NY 12180 USA; 3 Department of Materials Science and Engineering, University of Wisconsin-Madison, 1509 University Ave, Madison, WI 53706 USA; 4 Department of Chemical and Biological Engineering, Rensselaer Polytechnic Institute, 110 8th Street, Troy, NY 12180 USA; 5 Department of Materials Science and Engineering, Rensselaer Polytechnic Institute, 110 8th Street, Troy, NY 12180 USA; 6 U.S. Army Combat Capabilities Development Command Armaments Center, Watervliet Arsenal, NY 12189 USA; 7 Department of Electrical, Computer and Systems Engineering, Rensselaer Polytechnic Institute, 110 8th Street, Troy, NY 12180 USA

## Abstract

Unlike the vast majority of transition metal dichalcogenides which are semiconductors, vanadium disulfide is metallic and conductive. This makes it particularly promising as an electrode material in lithium-ion batteries. However, vanadium disulfide exhibits poor stability due to large Peierls distortion during cycling. Here we report that vanadium disulfide flakes can be rendered stable in the electrochemical environment of a lithium-ion battery by conformally coating them with a ~2.5 nm thick titanium disulfide layer. Density functional theory calculations indicate that the titanium disulfide coating is far less susceptible to Peierls distortion during the lithiation-delithiation process, enabling it to stabilize the underlying vanadium disulfide material. The titanium disulfide coated vanadium disulfide cathode exhibits an operating voltage of ~2 V, high specific capacity (~180 mAh g^−1^ @200 mA g^−1^ current density) and rate capability (~70 mAh g^−1^ @1000 mA g^−1^), while achieving capacity retention close to 100% after 400 charge−discharge steps.

## Abstract (web summary)

VS_2_ is a promising cathode material for lithium-ion batteries, but is susceptible to Peierls distortion during (de)lithiation. Here the authors show that VS_2_ cathodes can be stabilized by conformally coating them with a nanoscale TiS_2_ protective layer, leading to impressive electrochemical performance.

## Introduction

Transition metal dichalcogenides (TMDs) are an emerging class of two-dimensional (2D) materials with chemical formula of MX_2_, where M denotes a transition metal (e.g., Mo, W, Re, etc.) and X represents a chalcogen (e.g., S or Se)^1,2^. Recently there has been intense research activity directed towards deploying TMD materials for electrochemical energy storage applications^3–5^. However most of the commonly used TMDs such as molybdenum disulfide (MoS_2_) and tungsten disulfide (WS_2_) are semiconductors with low electrical conductivity, which limits their usefulness as battery materials. Deployment of such TMDs as electrodes is only possible when they are mixed with highly conductive carbon-based materials. However, this greatly reduces the active material loading and limits the high-rate capability of the electrode.

In contrast, vanadium disulfide (VS_2_) is a conducting TMD material^6^ and exhibits metallic behavior (Supplementary Fig. 1). Therefore, VS_2_-based electrodes could in principle be deployed in lithium (Li)-ion batteries without requiring a high content of conductive carbon additives or conductive binders, which are inactive materials. In spite of this promise, the realization of VS_2_-based electrodes^7–11^ in Li-ion batteries has been limited by its poor stability, which leads to low cycle life. In the literature, volume expansion leading to pulverization^7^ is cited as a reason for poor stability of VS_2_ in Li-ion batteries. To buffer the volume expansion, VS_2_ is mixed with mechanically resilient carbon materials (e.g. graphene) to create a composite electrode that offers improved stability^7^.

Here, we demonstrate that VS_2_ flakes can be stabilized in the electrochemical environment of a Li-ion battery by simply coating them with a few nm thick layer of titanium disulfide (TiS_2_). In our approach, densely packed VS_2_ flakes with high crystallinity are grown directly on the surface of a carbon nanotube current collector substrate by chemical vapor deposition (CVD). Then a conformal TiS_2_ coating is deposited on the VS_2_ platelets by atomic layer deposition (ALD). Electrochemical testing as well as in situ optical as well as ex situ scanning electron microscopy (SEM) observation reveal a pronounced stability enhancement for the TiS_2_-coated VS_2_ as compared to bare VS_2_. First-principles density functional theory (DFT) calculations indicate that unlike VS_2_ which undergoes large Peierls distortion during lithiation/delithiation, the TiS_2_ lattice remains relatively undisturbed. Consequently, the TiS_2_ coating provides an electrochemically and mechanically stable support that “buttresses” the VS_2_ platelets and prevents the underlying VS_2_ sheets from delaminating and peeling off the surface. To our knowledge, the role of Peierls distortion (during lithiation/delithiation) in promoting the failure of VS_2_ electrodes has not been identified in the past, and thus constitutes a key contribution of this work. The resultant VS_2_-TiS_2_ structures are remarkably resilient and offer a striking enhancement in stability over the baseline VS_2_ electrode in Li-ion batteries. In fact, the performance of the TiS_2_ protective coating is superior to ALD deposited layers of metal oxides (e.g. Al_2_O_3_, ZrO_2_, TiO_2_)^12^, fluorides (e.g. AlF_3_)^13^, and nitrides (e.g. TiN)^14^ that have been used to passivate different electrode surfaces. Deposition of such condensed and electrically insulating films induces low Li-ion diffusivity and electron transport, and thus suppresses initial capacity and rate capability^15^. In fact, such capacity quelling effects have been reported even for extremely thin (few angstroms thick) ALD deposited coatings. The TiS_2_ coating on the other hand does not suppress the specific capacity or the rate capability of VS_2_. This we presume is because TiS_2_ is also a TMD material and is compatible with VS_2_. Moreover, TiS_2_ is conductive and shares a similar electronic structure (Supplementary Fig. 1) as VS_2_, which allows the high-rate capability performance of VS_2_ to be retained despite being sandwiched by TiS_2_ layers.

## Results

### Synthesis and characterization

**Fig. 1:**
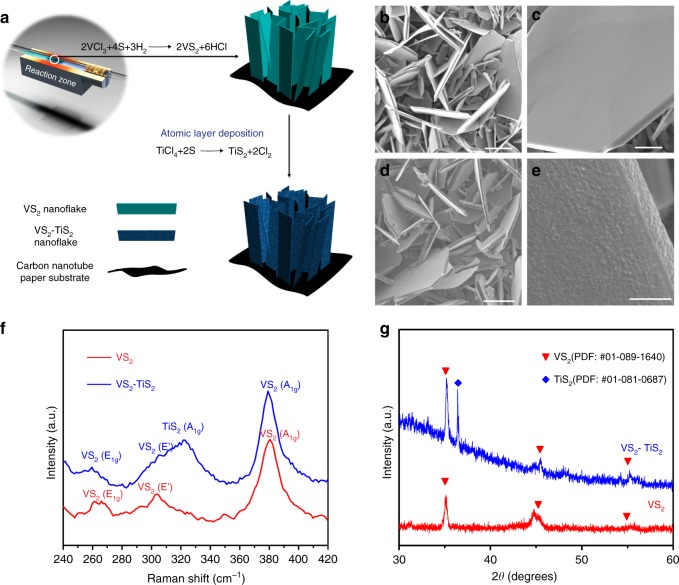
Synthesis and characterization of VS_2_ and VS_2_-TiS_2_ flakes. **a** Schematic of the procedure for fabricating VS_2_ and VS_2_-TiS_2_ composites. **b**, **c** Top-view SEM (**b**: scale bar = 10 μm; **c**: scale bar = 3 μm) of VS_2_ flakes with different magnification. **d**, **e** Top-view SEM (**d**: scale bar = 10 μm; **e**: scale bar = 1 μm) of the VS_2_-TiS_2_ structures with different magnification. **f** Raman spectrum of VS_2_ and VS_2_-TiS_2_. **g** X-ray diffraction pattern of VS_2_ and VS_2_-TiS_2_ flakes

A densely packed forest of VS_2_ sheets was grown (Methods) on a carbon nanotube current collector substrate (Supplementary Fig. 2) by a dual-zone atmospheric pressure chemical vapor deposition (APCVD) process (schematically represented in Fig. 1a). Since metal halide precursors^16^ tend to offer better growth regulation and reproducibility when compared to metal oxides, we have selected the chloride reactant (VCl_3_) and S powder as precursors for CVD growth. In addition, ~5% H_2_ was incorporated into the carrier gas in the CVD reactor in order to generate more active species (e.g., VCl^−^, VCl_2_^−^) from the vanadium precursor molecules. This enhances the nucleation site density and growth rate of the VS_2_ flakes. A carbon nanotube (CNT) film was selected as the current collector substrate because of its light weight, high conductivity and flexibility. The VS_2_ platelets are observed predominantly on the surface of the CNT film. An examination of the junction (Supplementary Fig. 3) between the VS_2_ and CNT substrate indicates that the VS_2_ platelets are embedded (or lodged) into the CNT current collector. This is important since it indicates a strong interface and good electrical connectivity between the VS_2_ flakes and the CNT current collector substrate.

After the VS_2_ growth was completed, ALD was used to deposit a nanolayered TiS_2_ coating over the VS_2_ platelets (VS_2_-TiS_2_) by using TiCl_4_ and S as precursors (Methods). The morphologies of the as-synthesized VS_2_ and VS_2_-TiS_2_ structures are revealed by top-view SEM imaging as shown in Fig. 1b−e. In Fig. 1b, densely packed micrometer scale VS_2_ flakes can be seen growing vertically off the current collector. The smooth flakes of regular hexagon or semi-hexagon shape (Fig. 1c) suggest their high crystallinity. The ALD-deposited TiS_2_ faithfully replicates the nanoforest structure and flake shape of the VS_2_ due to the excellent conformability and precise thickness control of the ALD technique (Fig. 1d). Note that there is a roughening (Fig. 1e) of the VS_2_ flakes post TiS_2_ deposition which is attributed to the polycrystalline phase of ALD TiS_2_. The elemental distribution results obtained by EDS elemental mapping (Supplementary Figs. 4–5) further confirm the homogeneous distribution of V, S and V, S, Ti atoms on the surfaces of VS_2_ and the VS_2_-TiS_2_ structures, respectively. X-ray photoelectron spectroscopy (XPS) also provided confirmation of the VS_2_ and TiS_2_ phase (Supplementary Fig. 6).

To evaluate the electrical conductivity of the VS_2_ and VS_2_-TiS_2_ flakes, electrical transport measurements were performed on individual flakes (Supplementary Fig. 7). Due to the two-dimensional morphology of the flakes, the sheet resistance^17^ was used to compare the conductivity of the VS_2_ and VS_2_-TiS_2_ flakes. The sheet resistance (*R*_s_) of the VS_2_ flake is in the 200–900 Ω ⎕^−1^ range, while that of the VS_2_-TiS_2_ flake lies in the 500–2400 Ω ⎕^−1^ range. After TiS_2_ deposition, the conductivity of the VS_2_ flake has decreased, but is still comparable to pure VS_2_. These results indicate that the intrinsic high electrical conductivity of VS_2_ is retained in the VS_2_-TiS_2_ architecture.

The VS_2_ and VS_2_-TiS_2_ flakes were further characterized by Raman spectroscopy (Fig. 1f). We observe Raman peaks of VS_2_ at 263, 304, and 379 cm^−1^. The peaks at 263 and 379 cm^−1^ correspond to the *E*_1g_ and *A*_1g_ modes of VS_2_ and are comparable to previous reports^16,18^ for CVD grown VS_2_. In our experiments, we also observed an additional peak at 304 cm^−1^, which we attribute to an in-plane sliding mode (*E*′) for VS_2_ that can arise due to interlayer mismatch^19^. ALD coating of TiS_2_ on VS_2_ introduced a new peak at 324 cm^−1^ which corresponds^20–22^ to the *A*_1g_ mode of TiS_2_. Other peaks exhibit similar position and width indicating that the VS_2_ structure was well preserved after the ALD process. X-ray diffraction (XRD) analysis also confirmed the crystallinity of the as-grown VS_2_ and VS_2_-TiS_2_ flakes (Fig. 1g). It should be noted that in the literature^7,16,18,23–27^ there is large scatter in the Raman data for VS_2_. This is illustrated in Supplementary Table 1. These differences arise due to the growth method (i.e., CVD vs. hydrothermal synthesis), laser (polarization) set-up, flake curvature as well as poor environmental stability when VS_2_ Raman measurements are taken under atmospheric conditions (Supplementary Fig. 8). These effects are discussed in detail in the Supplementary Information. Raman spectra of the CNT substrate before and after the depositions are provided in Supplementary Fig. 9. The G band at ~1580 cm^−1^ is attributed to vibration of sp^2^ bonded carbon atoms in the CNT, while the D-band (~1350 cm^−1^) is attributed to defects and disorder in the CNT^28^. The ratio of the intensity of the D to G band (*I*_D_/*I*_G_) of the pristine CNT film is ~0.45. This value is increased to ~0.6 after deposition. This slight increase in CNT defectiveness is presumably due to the high temperature and aggressive chemical environment related to the use of halogen precursors. There is also a higher frequency shoulder to the G band (D′ band ~1620 cm^−1^); this shoulder becomes more prominent after deposition. Also, a new peak at ~1438 cm^−1^ appeared post deposition, which is related to other carbonaceous materials^29^.

**Fig. 2:**
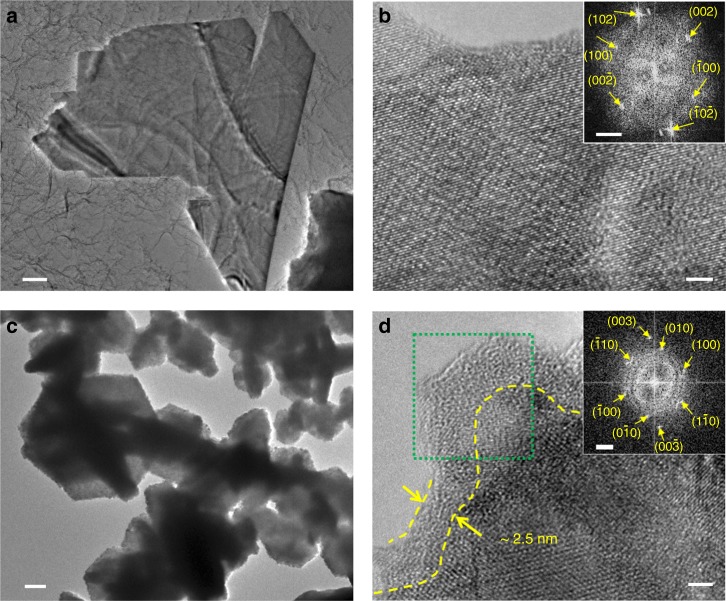
TEM and HRTEM characterization of VS_2_ and VS_2_-TiS_2_. **a** TEM (scale bar = 200 nm) and **b** HRTEM image (scale bar = 2 nm, inset shows the corresponding FFT pattern (scale bar = 2 nm^−1^)) of a typical VS_2_ flake. **c** TEM (scale bar = 200 nm) and **d** HRTEM image (scale bar = 2 nm, inset shows the FFT pattern (scale bar = 2 nm^−1^) of the region within the green box) of a typical VS_2_-TiS_2_ flake. TEM transmission electron microscopy, HRTEM high-resolution transmission electron microscopy, FFT Fast Fourier Transform

Transmission electron microscopy (TEM) was performed to probe the structure of the synthesized VS_2_ and VS_2_-TiS_2_ flakes. The flake edges appear to be roughened (Fig. 2a, c) after the TiS_2_ ALD deposition, which is consistent with the SEM observations in Fig. 1e. High-resolution TEM (HRTEM) imaging of VS_2_ is shown in Fig. 2b. The inset of Fig. 2b shows the corresponding fast Fourier transform (FFT) pattern. The well-defined spots in the FFT pattern indicates the single-crystal structure of the VS_2_ sheet planes viewed along the [010] zone axis. Some visible spots in the FFT are labeled as (102), $\left( {\bar 10\bar 2} \right)$, (002), $\left( {00\bar 2} \right)$, (100), and $\left( {\bar 100} \right)$. The measured reciprocal spacing *G*(100)_measured_ = 2.24 Å^−1^ using the calibration scale bar matches well with the calculated theoretical *G*(100)_theoretical_ = 2.25 Å^−1^. The real space spacing *d*_100_ = $(\sqrt 3 /2)$*a* = 2π/*G*(100), where *a* is the lattice constant. The extracted value of *a* ≈ 3.24 Å is consistent with the bulk lattice constant (3.221 Å) of VS_2_ (PDF: #01-089-1640). It is noted that weaker spots about 10° next to the (102) and $\left( {\bar 10\bar 2} \right)$ spots were observed in the FFT pattern. This is likely due to the rotation of the stacked multilayer VS_2_ structure during growth. Figure 2d shows HRTEM imaging of a VS_2_-TiS_2_ flake. ALD of TiS_2_ introduced a ~2.5-nm-thick layer on the surface. The inset in Fig. 2d is the FFT pattern from a selected region indicated by the green dashed box in the HRTEM image. Many spots from VS_2_ still can be identified, for example, (100), (010), $(1\bar 10)$ and (003), but there are more spots in ring-like contrast that is consistent with polycrystalline TiS_2_. FFT of other VS_2_-TiS_2_ flakes were analyzed and similar results were obtained. These confirm the existence of the TiS_2_ polycrystalline phase.

### Electrochemical properties

**Fig. 3:**
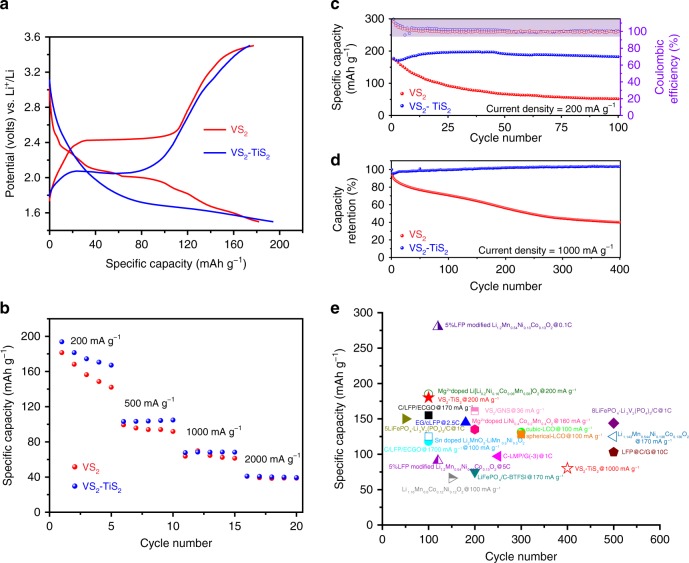
Electrochemical characterization. **a** The galvanostatic charge–discharge voltage profiles of the VS_2_ electrode and VS_2_-TiS_2_ electrode between 1.5 and 3.5 V (vs. Li/Li^+^) at a current density of 200 mA g^−1^. **b** Rate performance of the VS_2_ and VS_2_-TiS_2_ electrodes. **c** Cycling performance and Coulombic efficiency of VS_2_ electrode and VS_2_-TiS_2_ electrode at 200 mA g^−1^. **d** Capacity retention of VS_2_ electrode and VS_2_-TiS_2_ electrode at 1000 mA g^−1^. **e** Comparison of the VS_2_-TiS_2_ electrode (starred) with other commonly used cathodes [7, 37, 38, 39, 40, 41, 42, 43, 44, 45, 46, 47, 48, 49] in Li-ion batteries. The *y*-axis on the plot gives the specific capacity achieved by the cathode material at the completion of a specified number of charge−discharge cycles given on the *x*-axis of the plot. The charge−discharge rate (or current density) at which the electrode is cycled is also specified next to each data point on the plot

To evaluate the electrochemical performance of the VS_2_ and VS_2_-TiS_2_ electrodes, coin cells were assembled (Methods). The galvanostatic charge–discharge voltage profiles of the VS_2_ and VS_2_-TiS_2_ electrodes between 1.5 and 3.5 V (vs. Li/Li^+^) at a current density of ~200 mA g^−1^ are shown in Fig. 3a. The electrochemical intercalation and removal of Li in VS_2_ can be described by the following reaction: $x{\mathrm{Li}}^ + + xe^ - + {\mathrm{VS}}_2 \leftrightarrow {\mathrm{Li}}_x{\mathrm{VS}}_2$. The voltage plateau in the discharge/charge curve at about 2 V is indicative of the transition from the α-VS_2_ to the β-Li_*x*_VS_2_ phase^7^. The lithiated Li_*x*_VS_2_ phase shows promise as a cathode material in Li-ion batteries. It is noteworthy that the TiS_2_ surface coating does not suppress the specific capacity of VS_2_. As Fig. 3a indicates both the bare VS_2_ and the VS_2_-TiS_2_ core-shell electrode delivers a similar initial (i.e. first cycle) specific capacity of about 180 mA h g^−1^. For the VS_2_ electrode, the mean operating voltage is at ~2.3 V. After TiS_2_ coating, the operating voltage of the VS_2_-TiS_2_ electrode dropped to ~2.1 V. One reason for this might be the lattice stress caused by TiS_2_ growth on the surface of the VS_2_ flake, which may affect the intercalation and de-intercalation potential of lithium ions into VS_2_. Moreover, it has been commonly observed that ALD-coated layers affect lithium diffusion and electron transport processes^12,30^, which could also influence the charge−discharge voltage profiles.

The rate performances of the VS_2_ and VS_2_-TiS_2_ electrodes was investigated as shown in Fig. 3b. Since TiS_2_ is metallic (Supplementary Fig. 1) similar to VS_2_, the conductivity of VS_2_-TiS_2_ is still comparable to that of pure VS_2_ (Supplementary Fig. 7). Consequently, the overall rate performance of the VS_2_ electrode was not significantly affected by the TiS_2_ coating. The results show that the baseline VS_2_ and the VS_2_-TiS_2_ electrode exhibits a capacity above 150 and 180 mA h g^−1^, respectively in the first five cycles at a current density of ~200 mA g^−1^. Even when the current density is increased to ~2000 mA g^−1^ (~10C charge−discharge rate), a specific capacity of ~40 mA h g^−1^ is achieved, which indicates that the VS_2_-TiS_2_ electrode is capable of high C-rate operation. The cyclic stability of the samples was investigated at a current density of ~200 mA g^−1^, as shown in Fig. 3c. The VS_2_-TiS_2_ electrode demonstrated excellent cycle stability above 180 mA h g^−1^ after 100 charge−discharge steps, while the baseline VS_2_ electrode under the same conditions exhibits a rapid capacity loss. We also investigated the cycling performance of the electrodes at a higher current density of ~1000 mA g^−1^ (see Fig. 3d). The capacity retention rate is close to ~100% for the VS_2_-TiS_2_ electrode after 400 continuous cycles of charge and discharge, while the capacity retention of the baseline VS_2_ electrode was only ~40% under the same test conditions. The charge and discharge voltage profiles for the VS_2_ and VS_2_-TiS_2_ electrodes for the 1st, 100th, 200th, 300th and 400th cycles are provided in Supplementary Fig. 10. From the charge−discharge profiles, it is evident that the stability of the electrodes has been significantly improved by the TiS_2_ coating. Moreover, the over-potential between charge and discharge did not amplify with cycling in the VS_2_-TiS_2_ electrode, which is quite different from the pristine VS_2_ electrode for which the over-potential continues to increase with cycling.

Figure 3e compares the VS_2_-TiS_2_ performance with the other cathodes that are commonly used in Li-ion batteries. At low current densities (~200 mA g^−1^), the VS_2_-TiS_2_ electrode provides a very high specific capacity of over 180 mA h g^−1^ (after 100 charge−discharge cycles) which is among the best results reported to date. Moreover, unlike many of the other cathode materials, the VS_2_-TiS_2_ electrode can also be operated at high charge−discharge rates and provides a capacity of ~70 mA h g^−1^ (after 400 cycles) at a current density of ~ 1000 mA g^−1^. In the reported literature, only lithium iron phosphate (LFP)-based cathodes provide higher specific capacities at comparable charge−discharge rates. Note that these results are reported for a realistic mass loading (~ 3.5 mg cm^−2^) of the VS_2_-TiS_2_ material.

### In situ optical characterization

**Fig. 4:**
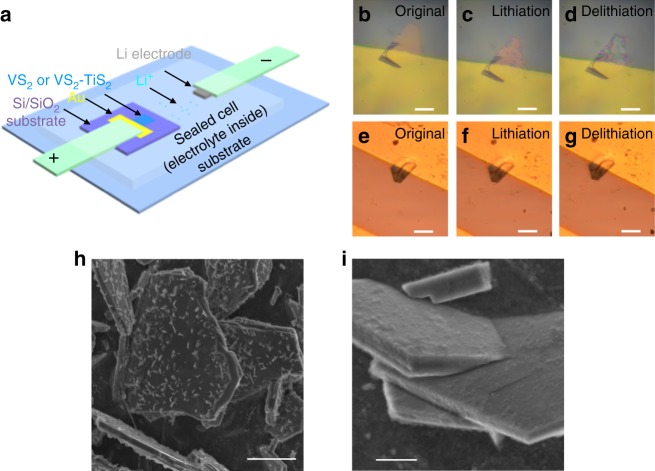
Observations of structural changes to the VS_2_ and VS_2_-TiS_2_ flakes during the lithiation and delithiation process. **a** Schematic of transparent cell set-up for in situ optical imaging. **b**−**d** Optical images of VS_2_ flake at the original status, lithiation status, and delithiation status. Scale bar = 20 μm. **e**−**g** Optical images of VS_2_-TiS_2_ flake at the original status, lithiation status, and delithiation status. Scale bar = 20 μm. The color difference of the background shown in (**b**−**d**) and (**e**−**g**) was caused by using a different light source of the optical microscope. **h** Ex situ SEM image (scale bar = 10 μm) of VS_2_ flakes after galvanostatic charge−discharge cycling at 1000 mA g^−1^ for 400 cycles. **i** Corresponding SEM image (scale bar = 3 μm) of VS_2-_TiS_2_ flakes after galvanostatic charge−discharge cycling at 1000 mA g^−1^ for 400 cycles. SEM scanning electron microscopy

In order to monitor structural changes during lithiation and delithiation, we carried out in situ observation of an individual VS_2_ and VS_2_-TiS_2_ flake during the charge−discharge process. The schematic of the optical transparent cell is shown in Fig. 4a with the VS_2_ (or VS_2_-TiS_2_ flake) as the device cathode and Li metal as the anode. The cell was sealed using a transparent glass cover (Supplementary Fig. 11), through which in situ observation of structural changes during lithiation and delithiation could be performed. To better monitor the structure change, we chose relatively thinner flakes of ~20 nm in thickness (Supplementary Fig. 12). The freshly sealed VS_2_ or VS_2_-TiS_2_ flake showed an open circuit voltage at around 2.6 V vs. Li/Li^+^. We carried out lithiation by gradually decreasing the cathode potential to 1.5 V (vs. Li/Li^+^). Subsequently, the cathode potential was increased to 3.5 V (vs. Li/Li^+^), to complete the delithiation process. Voltage profiles for the VS_2_ and VS_2_-TiS_2_ cells are shown in Supplementary Fig. 13. Note that the gap between the electrodes is much larger for the in situ cell as compared to a coin cell, which lead to a higher over-potential (Supplementary Fig. 13).

As shown in Fig. 4b−d, for the pure VS_2_ flake, we observe significant changes in the color and morphology of the flake under the optical microscope. During the lithiation process (Fig. 4c), partial transparency appeared on the original VS_2_ flake, and this change was not reversible when carrying out the delithiation process. In fact, the transparency spreads over the entire VS_2_ flake, indicating significant permanent (irreversible) change or damage to the flake. In stark contrast to this, with the TiS_2_ coating, there is no obvious color or structural change to the VS_2_-TiS_2_ flake as shown in Fig. 4e−g. The reflection light intensity contrast, which is the reflection from the 2D flake compared with that from the substrate, is related to the absorption of the 2D flake. The observed transparency spreading can thus be understood as a decrease in the overall absorption of the VS_2_ flake. Since uncoated VS_2_ is not stable during lithium intercalation, it likely undergoes delamination, which effectively decreases the overall absorption and hence induces the observed transparency. This interpretation is confirmed by the completely different behavior of TiS_2_-coated VS_2_. The TiS_2_-coated VS_2_ is stable during the lithium intercalation process and we expect the thickness of the VS_2_ flake remains unchanged. The reflection contrast of the TiS_2_-coated VS_2_ flake, therefore, remains unchanged during and after the lithium intercalation, consistent with our observation.

### Ex situ SEM characterization

These results were also corroborated by ex situ SEM imaging of the flakes. For this, we opened the coin cells (tested in Fig. 3d) that were cycled for 400 charge−discharge steps inside a glove box. The VS_2_ and VS_2_-TiS_2_ electrodes were extracted from the coin cells, and SEM was used to observe the morphology of these electrodes. As shown in Fig. 4i, no obvious change in the morphology can be observed for the VS_2_-TiS_2_ electrode. These observations are consistent with the in situ optical imaging and indicate that with the help of the TiS_2_ coating, the structural integrity of the VS_2_ could be maintained during electrochemical cycling. In contrast, SEM imaging of the bare VS_2_ electrode revealed a multitude of VS_2_ flakes delaminating and peeling off (see Fig. 4h) the surface of the electrode, confirming structural damage during cycling, which is responsible for the fast capacity fade.

### First principles calculations

**Fig. 5:**
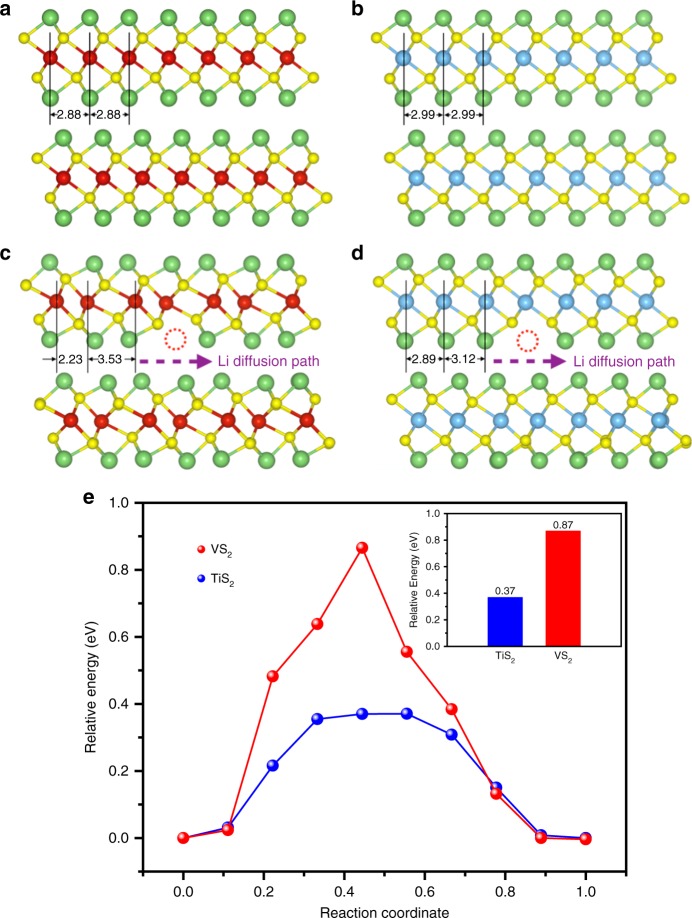
DFT calculations. Fully intercalated pristine layered structures, **a** Li_2_VS_2_ and **b** Li_2_TiS_2_. Here, the yellow and green balls symbolize sulfur and lithium atoms, respectively. Vanadium atoms are red and titanium atoms are blue. Lithium diffusion in these systems requires that a neighboring lithium site is vacant. The corresponding intercalated structures with Li vacancies, marked with dashed red circles are shown for **c** Li_2_VS_2_ and **d** Li_2_TiS_2_. Lithium diffusion paths are indicated by purple arrows. Notice that the even spacing of 2.88 Å between vanadium atoms in fully intercalated Li_2_VS_2_ distort to 2.23 and 3.53 Å when a lithium vacancy is introduced. The distortion is much smaller in Li_2_TiS_2_, for which the distances between titanium atoms distort from 2.99 Å to 2.89 Å and 3.12 Å. **e** Energy profile along the lithium diffusion path in the VS_2_ and TiS_2_ (inset indicates the energy barriers for lithium migration). Reaction coordinates 0 and 1 correspond to the beginning and end of the lithium migration paths, which are equilibrium systems, while noninteger reaction coordinates in-between denote the fraction of the lithium migration path that has been traversed. DFT density functional theory

Density functional theory calculations were carried out (Methods) to obtain a fundamental understanding of lithiation/delithiation in and out of the VS_2_ and VS_2_-TiS_2_ structures. The calculations are not for a single sheet, rather all calculations assumed a bulk structure. That is, the out-of-plane lattice constants were chosen so that it minimized the free energy of bulk Li_2_VS_2_ and bulk Li_2_TiS_2_. The dynamics of an individual Li-ion can be described as a series of migrations between adjacent transition metal sites belonging to the same MS_2_ (M = V or Ti) layer. Each of these migrations requires that an adjacent Li site be vacant. It therefore follows that the migration of a Li-ion is also the migration of a vacancy in the opposite direction. To model these migrations, we remove a Li-ion from a fully intercalated system, leaving behind a single Li vacancy. We then use the climbing-image nudged elastic band method (CNEB) to calculate the energy barrier associated with moving a Li-ion from an adjacent site into that vacancy. Figure 5e compares the energy profiles of these migration processes in VS_2_ and TiS_2_. Clearly, VS_2_ is associated with a much larger energy barrier for migration. This is because VS_2_ and TiS_2_ respond very differently to the presence of a Li vacancy. Specifically, VS_2_ layers “Peierls distort” when a vacancy is introduced, so that the V atoms are alternatingly pinched and pulled apart (Fig. 5a, c). When a Li migrates, the VS_2_ distortion must reverse its phase, i.e., the pinched atoms separate, while the pulled atoms converge. This rearrangement has an energy cost which is reflected in VS_2_’s tall energy profile (Fig. 5e). Meanwhile, the fully intercalated TiS_2_ crystal is much less susceptible to distortion, in that we see very little deviation from the pristine structure when a vacancy is introduced (Fig. 5b, d). As a result, the TiS_2_ crystal remains relatively undisturbed during the migration process. This can explain why the TiS_2_ coating dramatically improves the lifetime of the VS_2_ cathode. Through repeated charging and discharging cycles, periodic agitation to the host VS_2_ material can cause degradation over time. With TiS_2_ coating, the robust outermost layers endure much less disturbance during Li migration. As a result, the outer TiS_2_ is resilient to the charging/discharging cycles, while the inner VS_2_ layers are more protected from degradation due to the mechanical support provided by the TiS_2_ coating.

To extend the generality of our study, we also investigated partially intercalated systems (Supplementary Fig. 14), which contains one Li per unit of VS_2_ or TiS_2_. We found that LiVS_2_ Peierls distorts in a manner similar to what we found in Li_2_VS_2_. Meanwhile, the distortion in LiTiS_2_ is imperceptible (Supplementary Fig. 14), as it was in Li_2_TiS_2_. Note that the cathode likely contains regions of both LiVS_2_ and Li_2_VS_2_, resulting in an intermediate capacity as observed in the experiments. These results suggest that the distortion of Li-intercalated VS_2_ appears at various intercalation concentrations, and therefore occurs throughout the entire charge/discharge process. By contrast, Li-intercalated TiS_2_ remains resilient to distortion, suggesting that the TiS_2_ coating can protect the inner VS_2_ through the complete charge−discharge cycle.

Since DFT indicates that TiS_2_ offers superior stability compared to VS_2_, then would a bulk TiS_2_ flake be superior to VS_2_-TiS_2_? While TiS_2_ is a metal, it is ~4.5 times less conductive than VS_2_. Reported electrical conductivity value for VS_2_^18^ is ~3000 S cm^−1^ compared to ~676 S cm^−1^ for TiS_2_^31^. For ~2.5 nm TiS_2_ coating, this is not a significant limitation (Supplementary Fig. 7), but for thicker TiS_2_ flakes, the lower conductivity can limit performance, especially for fast charging and high power applications. For this reason, the VS_2_-TiS_2_ electrode is preferred to only TiS_2_ or only VS_2_.

Lastly, we used DFT to investigate the binding strength of lithium with pure VS_2_ and VS_2_ with a coating layer of TiS_2_. Our calculations indicate that the binding energy of lithium to the second layer of fully charged, pure VS_2_ is 3.12 eV. By replacing the top layer of VS_2_ with TiS_2_, this binding energy is slightly reduced to 3.09 eV. Assuming that binding energies should result in more residual lithium trapped in the material after the recharging process, we can use these energies to estimate the ratio of lithium occupation in both materials after delithiation. The canonical distribution of statistical mechanics predicts this ratio to be: ${{R}} = e^{ - \Delta E/k_{\mathrm{B}}T}$, where Δ*E* is the difference in formation energies, *k*_B_ = 8.62 × 10^−5^ eV K^−1^ is Boltzmann constant, and *T* = 293 K is the room temperature. Inserting Δ*E* = 3.13 − 3.09 = 0.03 eV, we estimate that the residual lithium occupation in pure VS_2_ to be about three times greater than that of VS_2_ with the TiS_2_ coating. As a result, we expect that delithiation, and therefore the recharging of the battery, is more complete with the TiS_2_ coating. This result also indicates that the presence of TiS_2_ improves the capacity retention of the battery during charge−discharge cycles.

## Discussion

To summarize, we have demonstrated a procedure to stabilize VS_2_ cathodes in Li-ion batteries, based on coating with TiS_2_. Electrochemical testing, in situ optical imaging and first principles DFT calculations indicate that the stability of the battery is drastically improved after the VS_2_ core is encapsulated by a thin (~2.5 nm) TiS_2_ coating layer. SEM imaging of the VS_2_ electrode post cycling indicates that VS_2_ layers tend to delaminate (i.e., peel off) the surface of the bulk VS_2_ flake. This is to be expected as structural distortion due to Li intercalation and extraction will have the greatest negative impact on the outermost VS_2_ layers. These layers are the least protected and the most vulnerable. The VS_2_ sheets within the bulk of the flake are mechanically supported (buttressed) by neighboring sheets and are therefore far less likely to detach or separate from the flake. Therefore supporting the outermost VS_2_ layers close to the surface becomes critical, which is what the ~2.5 nm-thick TiS_2_ coating is able to accomplish. Since there is minimal lattice distortion to the TiS_2_ coating, the TiS_2_ layer remains intact on the VS_2_ flake. Its presence prevents the underlying VS_2_ layers from delaminating from the surface of the flake, in spite of the large lattice distortion and volume changes that these underlying layers will encounter on lithiation and delithiation. In this way, the presence of TiS_2_ at the surface preserves the structure of VS_2_ and prevents the delamination and break-up of the VS_2_ material during the lithiation−delithiation process. These findings provide new opportunity for the rational design of conductive TMD materials for building high-performance Li-ion batteries.

## Methods

### VS_2_ growth on the carbon current collector by CVD

Dual-zone APCVD was utilized for the synthesis of vanadium disulfide (VS_2_) sheets on the carbon nanotube current collector. As shown in schematic Fig. 1a, the source boats containing vanadium trichloride (VCl_3_) and sulfur (S) powders were arranged within a quartz tube in the furnace tube, where VCl_3_ was located at the center of the furnace, while S was placed ~25 cm upstream. The substrate which is about 14 mm × 75 mm × 0.02 mm (W × L × H) faced downward on the top edge of the source boat containing VCl_3_. Since the ratio between vanadium and sulfur was critical to achieving the suitable stoichiometry, the evaporation of VCl_3_ and S powders were independently controlled by two temperature zones. In the optimized growth condition, a quartz boat containing ~0.1 g VCl_3_ powders (Sigma-Aldrich) was placed in the center of the first furnace zone, and a quartz boat containing ~0.6 g S powders (Sigma-Aldrich) was positioned in the center of the second furnace zone. Both boats were semi-cylindrical cuts from a quartz tube without vertical edges along the direction of flow, which allows for more even downstream flow of the precursor materials to the substrates. Before the growth, the quartz tube was baked at ~120 °C and purged with ~80 sccm forming gas (nitrogen mixed with ~5% hydrogen) for ~1 h. Then, the heating zone 1 with the VCl_3_ boat was heated to ~750 °C at a rate of ~80 °C/min. The temperature of the S boat during growth was ~250 °C. When the VCl_3_ temperature reached ~750 °C, the gas flow was reduced to ~50 sccm. After ~10-min growth of VS_2_ under atmospheric pressure, the flow rate was increased to ~80 sccm again and the system was naturally cooled down to room temperature over ~2.5 h.

### TiS_2_ ultrathin layer coating by ALD

The VS_2_ sample was loaded in the ALD chamber for polycrystal TiS_2_ coating at ~400 °C. A solid-precursor-cylinder containing S powder was connected to the ALD system for sulfur supply, while titanium tetrachloride (TiCl_4_) was used as the titanium (Ti) precursor. To maintain the high vapor pressure of both precursors, the temperature of the solid-precursor-cylinder and TiCl_4_ cylinder were maintained at ~400 °C and ~30 °C, respectively. The base pressure of the system was ~900 mTorr by using Ar as the carrier gas (~100 sccm). The ALD growth cycle consisted of ~10 s S pulsing + ~30 s Ar purging + ~5 s TiCl_4_ pulsing + ~30 s Ar purging. One hundred cycles yielded ~2 nm thick polycrystal TiS_2_ coating (VS_2_-TiS_2_) with the deposition rate of ~0.02 nm/cycle.

### Surface and crystal structure characterizations

The structure of the materials (VS_2_-TiS_2_ and VS_2_) was investigated by SEM (Carl Zeiss Supra field-emission scanning electron microscope). Energy-dispersive X-ray spectroscopy (EDS) in conjunction with SEM analysis and Raman spectroscopy measurements were conducted to obtain the surface elemental information. The Raman measurements were performed with a lab-made micro Raman spectrometer. To prevent possible oxidation of vanadium disulfide caused by laser irradiation, the samples were mounted in an optical cryostat covered by a quartz window with an internal pressure of ~7×10^−7^ Torr. The 532 nm laser with a spot size of ~2 μm and intensity of ~5 mW (LRS-0532 DPSS, Laserglow Technologies, Canada) was irradiated on the sample through the quartz window. The reflected signal was obtained and analyzed by a high-speed spectroscopy CCD camera (iDus 420 Series, Andor Technology Ltd, UK) and imaging spectrometer (Shamrock 500i, Andor Technology Ltd, UK). The resolution of the Raman system is ~0.6 cm^−1^. The step size used in measurements was ~1.34 cm^−1^. All spectra were collected by integrating the signal for ~60 s. A Tecnai TF-30 transmission electron microscope (FEI, Hillsboro, OR) was used to study the crystal structure of synthesized flakes and the surface distribution of TiS_2_ layer. Focused ion beam (FIB) imaging was performed on an FEI Helios Nanolab 600i dual beam electron microscope. The FIB was conducted with gallium ions operating at 30 kV and electron imaging was performed at 15 kV. XPS was carried out using Al Kα radiation (~1486 eV) in a PHI 5000 Versaprobe^TM^ system with a hemispherical analyzer and an 8-channel detector. XRD was done in a PANalytical X’Pert Pro Diffractometer.

### Electrochemical measurements

The electrochemical properties of the VS_2_ flakes without/with TiS_2_ coating as cathode materials in Li-ion batteries were evaluated by a galvanostatic charge/discharge technique. Coin cells (2032-type) were used to assemble test cells in an Ar-filled glove box (MBraun Labstar). The electrodes (VS_2_, VS_2_-TiS_2_) and current collector were cut to the desired shape without binder or additional conductive additives. The mass loading of the composite was ~3.5 mg cm^−2^ and the capacity was normalized on the total mass of the electrodes. Metallic lithium was used as the counter/reference electrode. A total of ~30 μL of ~1 M lithium bis(trifluoromethanesulfonyl)imide in 1,3-dioxolane and 1,2-dimethoxyethane (1:1 by volume) with ~0.1 mol L^−1^ LiNO_3_ additive was used as the electrolyte. Charge−discharge measurements were carried out galvanostatically at various current densities over a voltage range of 1.5 to 3.5 V (vs. Li/Li^+^) using an Arbin BT2000 battery testing system.

### In situ optical observation

A designed transparent cell was built for in situ optical observation during the lithiation−delithiation process. For this, the VS_2_ flakes grown by APCVD were transferred onto an SiO_2_/Si substrate (~300 nm SiO_2_) by simple tapping. VS_2_ nanoflakes on the substrate with a lateral size of ~30 µm were targeted by optical microscopy. Next a shadow mask with grids of ~80 µm were applied on the top to deposit the Au pattern by e-beam evaporation, so as to partially cover the flake. The patterned sample that acts as the cathode in the Li-ion cell was then transferred into the glove box for the device assembly. A Li anode, the patterned VS_2_ or VS_2_-TiS_2_ cathode and the liquid electrolyte were encapsulated within a transparent enclosure. The anode and cathode materials were in contact with stainless steel meshes that served as the current collectors for the battery. A cover glass was placed on top of the central region and the electrolyte was filled into this region. The visualization cell, including cut outs for the current collectors were sealed using epoxy (Supplementary Fig. 11).

### Density functional theory simulation

Calculations utilized plane-wave DFT with projector augmented-wave (PAW) pseudopotentials as implemented in the Vienna ab initio Simulation Package (VASP)^32^. The generalized gradient approximation (GGA) was employed using the Perdew−Burke−Ernzerhof functional^33^, for which van der Waals interactions were considered using density functional methods of optB86 ^34,35^. Ionic relaxation calculations used a plane-wave basis set including energies up to 550 eV. We assume that, when fully lithiated, the bulk TMD crystals can hold two Li for each unit of MS_2_ (M = Ti or V), with one Li bound above and one below each transition metal atom. Hence, we denote the intercalated TMD by the chemical formula Li_2_MS_2_. For the primitive bulk cells of Li_2_VS_2_ and Li_2_TiS_2_, the Brillouin zone was sampled with a Γ-centered Monkhorst−Pack mesh of dimensions 11 × 11 × 4. Relaxation iterations persisted until all interatomic Hellmann−Feynman forces settled below 0.01 eV Å^−1^ for all atoms, while the self-consistent field iterations ceased when the changes in both total energy and the eigenvalues settled below 10^−6^ eV between iterations. Calculations involving cathode surfaces included 16 Å of vacuum separation to eliminate interactions between periodic adjacent cells. Larger amounts of vacuum produced nearly identical ground state energies.

During the discharging process, the intercalated Li evacuate the crystal, traveling in-plane between the MS_2_ layers towards the crystal’s edge via successive migrations to unoccupied Li sites. The activation energies required for these migration were calculated using the nudged elastic band method combined with the climbing-image technique^36^. For these calculations, a Li vacancy is placed in an otherwise fully intercalated 4 × 4 × 2 supercell, while eight intermediate images are used to describe its migration to an adjacent site.

In addition, the formation energies of dilithiation of a single layer were calculated using; *E*_*form*_ = *E*_int_ − *E*_vac_ − 2 × *E*_Li_, where *E*_int_, *E*_vac_, and *E*_Li_ are the free energies of a fully intercalated system, a system with one layer of Li removed, and an isolated Li^+^ ion. The binding energies are then the additive inverses of the free energies.

Lastly, to extend the generality of our study, we investigated lithiation and delithiation in partially intercalated TMDs, which contains one Li per unit of MS_2_ and is denoted as LiMS_2_. We removed one Li from an otherwise pristine 4 × 4 × 2 LiMS_2_, and allowed the system to relax, respecting the same convergence criteria as described above.

## Supplementary information

Supplementary Information

## Data Availability

All relevant data are available from the corresponding author upon request.
