# Supplementary material for: Vanadium disulfide flakes with nanolayered titanium disulfide coating as cathode materials in lithium-ion batteries
Source: Nat Commun. 2019 Apr 16;10:1764. doi: 10.1038/s41467-019-09400-w (PMC6467906; doi:10.1038/s41467-019-09400-w)
Supplement: Supplementary file 1 — Supplementary Information [file 41467_2019_9400_MOESM1_ESM.pdf]

SUPPLEMENTARY INFORMATION

Vanadium disulfide flakes with nanolayered titanium disulfide coating as cathode materials in lithium ion batteries

Li et al.

Supplementary Figures

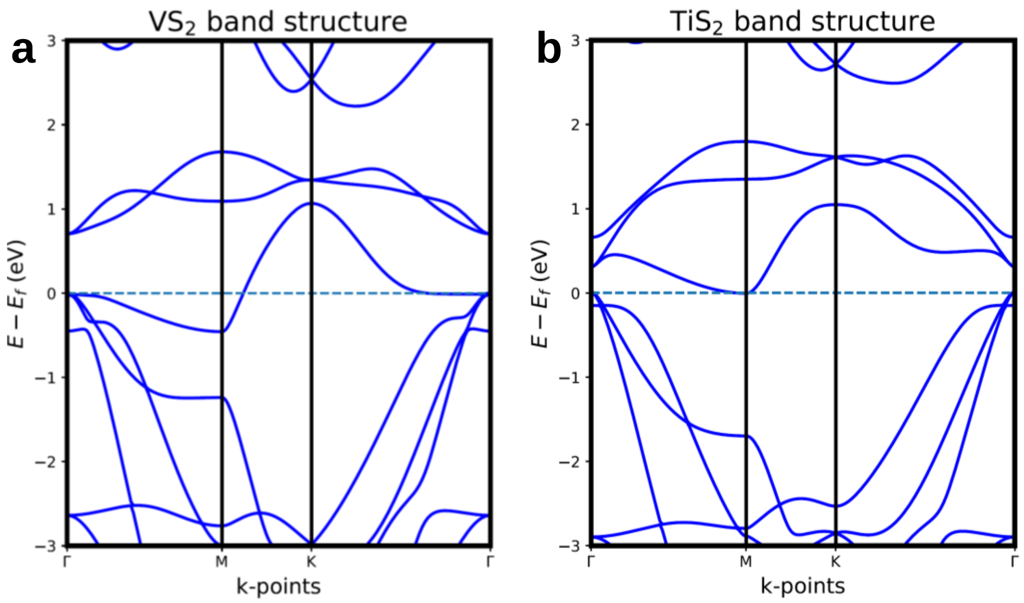

Supplementary Figure 1. DFT band structure calculations. Calculated band structures of (a) VS_2 and (b) TiS_2.

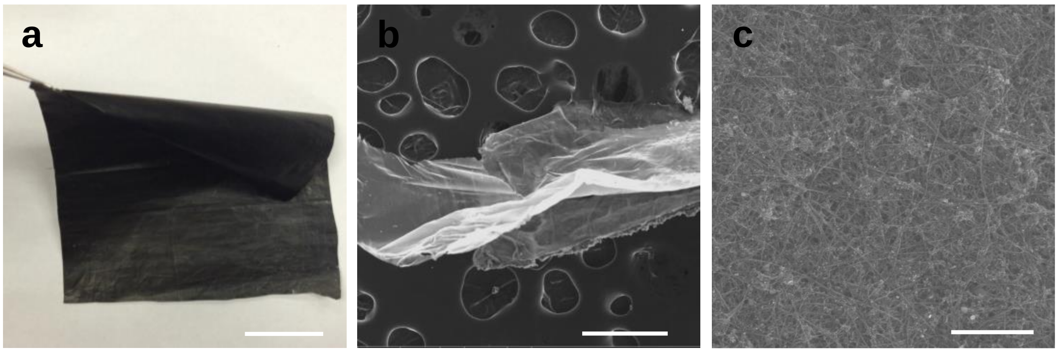

Supplementary Figure 2. Carbon nanotube current collector. (a) Digital photo (scale bar = 4 cm) (b) low magnification SEM (scale bar = 400 μm) and (c) high magnification SEM (scale bar = 1 μm) of the flexible carbon nanotube current collector substrate.

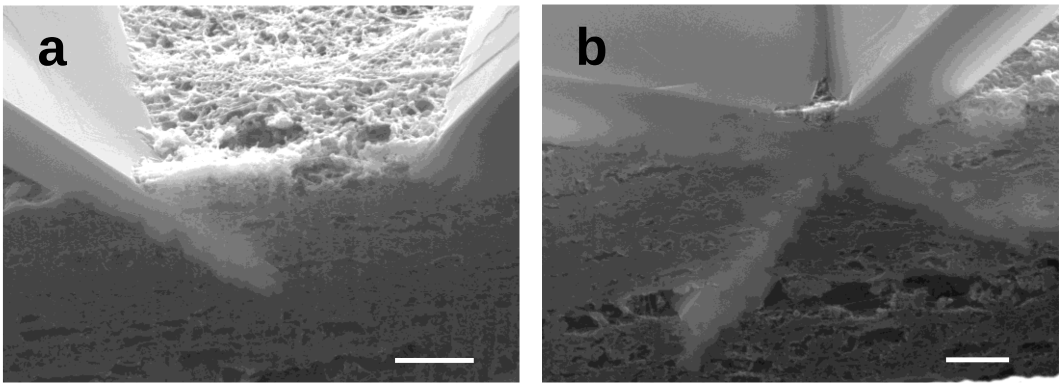

Supplementary Figure 3. Imaging of the interface between the flake and the current collector substrate. (a-b) SEM imaging (scale bar = 1 μm) of the cross-section of the CNT substrate indicating VS_2 flakes lodged into the top surface of the CNT current collector. Focused ion beam (FIB) was used to section the CNT substrate.

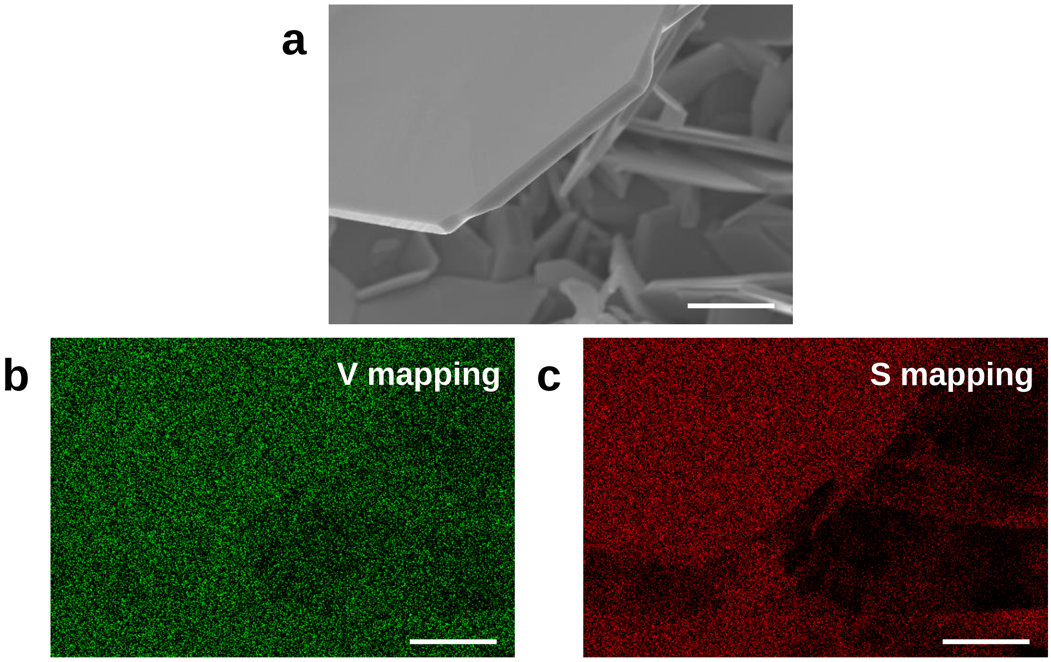

Supplementary Figure 4. SEM imaging and elemental mapping of VS_2. (a) Top-view SEM image of the VS_2 electrode and (b) vanadium and (c) sulfur elemental mapping results from (a). Scale bar = 5 μm.

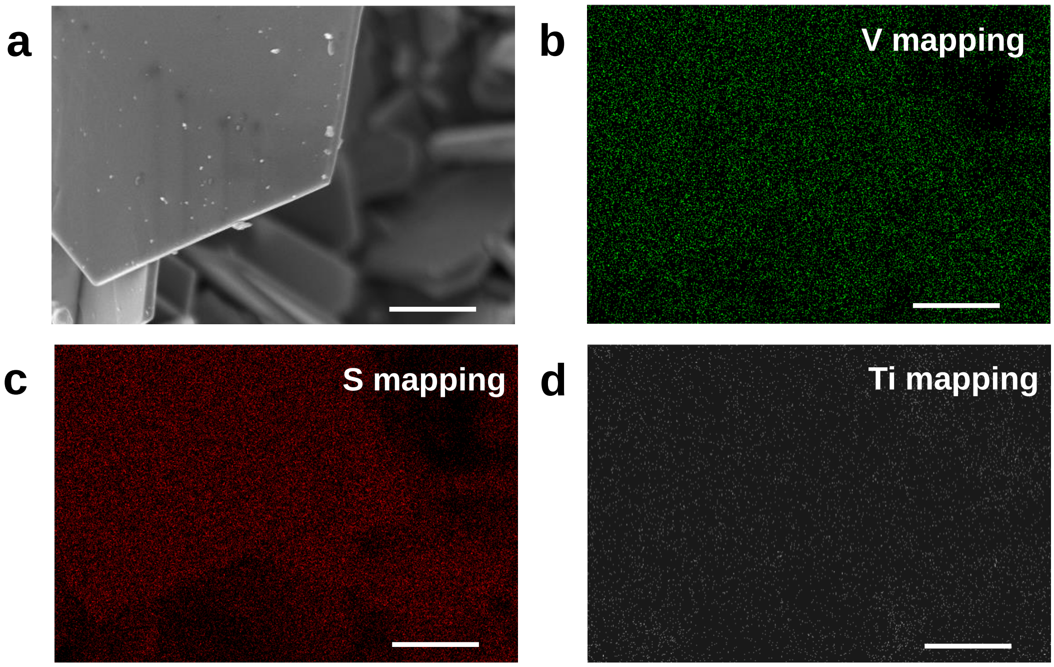

Supplementary Figure 5. SEM imaging and elemental mapping of VS_2-TiS_2. (a) Top-view SEM image of the VS_2-TiS_2 electrode and (b) vanadium, (c) sulfur and (d) titanium elemental mapping results from (a). Scale bar = 5 μm.

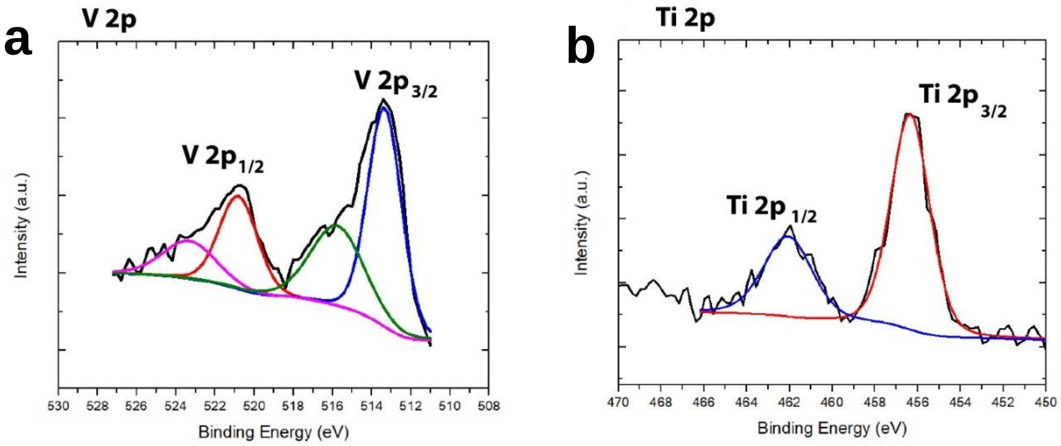

Supplementary Figure 6. XPS characterization. High-resolution XPS scans of (a) V 2p and (b) Ti 2p from the as-grown flakes. The energy positions of the V-peaks, which correspond to 513.5 eV and 521.1 eV (V $2p_{3/2}$ and V $2p_{1/2}$), indicate a valence of +4, corresponding to the VS_2 phase[1,2]. The peak positions for Ti $2p_{3/2}$ and Ti $2p_{1/2}$ at 456.10 eV and 462.20 eV correspond[3] to titanium in TiS_2.

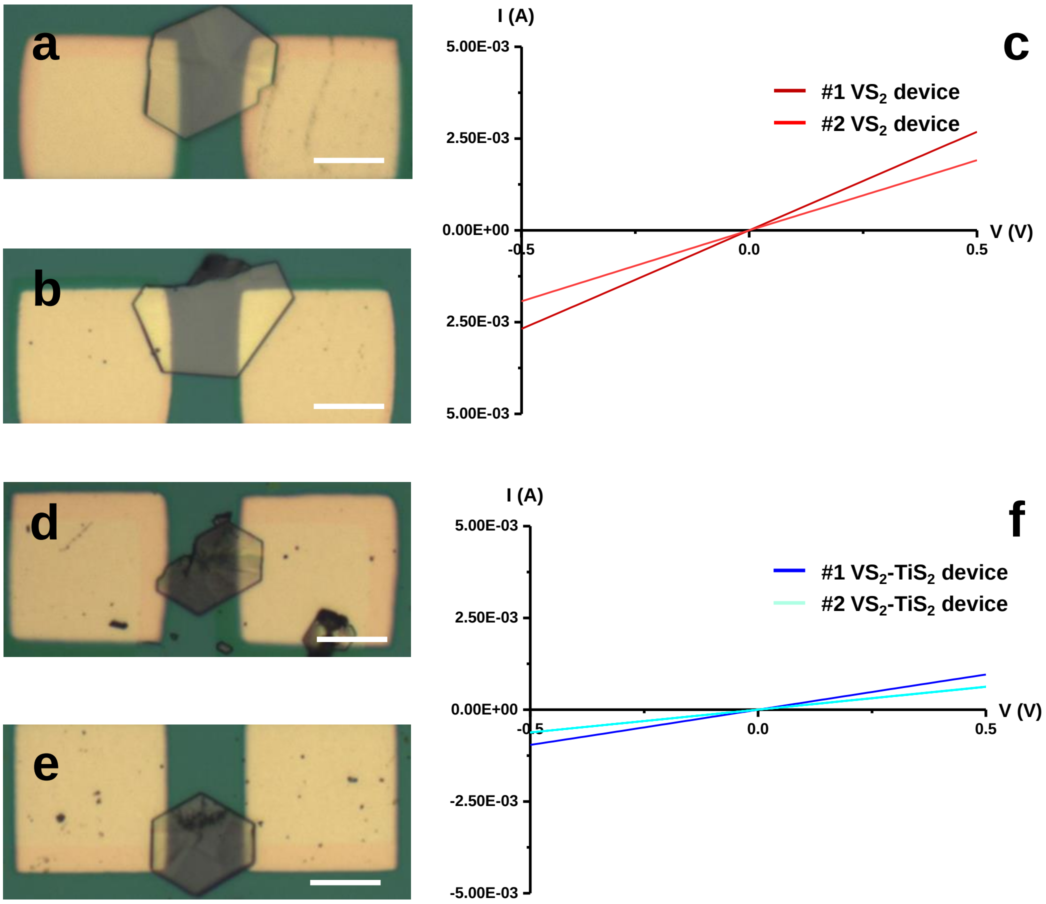

Supplementary Figure 7. Electrical conductivity measurements. (a, b) VS_2 flake with metal (Au) contacts. (c) Current-Voltage curve of (a) and (b). (d, e) VS_2-TiS_2 flake with Au contacts. (f) Current-Voltage curve of (d) and (e). By varying the applied voltage between the two Au contacts, we obtained the current-voltage response and the flake resistance (R) as shown in (c, f). Due to the two-dimensional morphology of the flakes, the sheet resistance (R_s) is expressed as:

$$R_S = \mathrm{R} \times \frac{width}{length}$$

where, R is calculated from the current-voltage curves and the aspect ratio range of the flakes is about 1 to 3. R_s is used to evaluate and compare the conductivity of the VS_2 and VS_2-TiS_2 flakes. R_s of the VS_2 flake is about 200-900 $\Omega\ \square^{-1}$ while that of the VS_2-TiS_2 flake is about 500-2400 $\Omega\ \square^{-1}$. After TiS_2 deposition, the conductivity of the VS_2 flake has decreased, but is still comparable to pure (i.e., uncoated) VS_2. Scale bar = 20 μm.

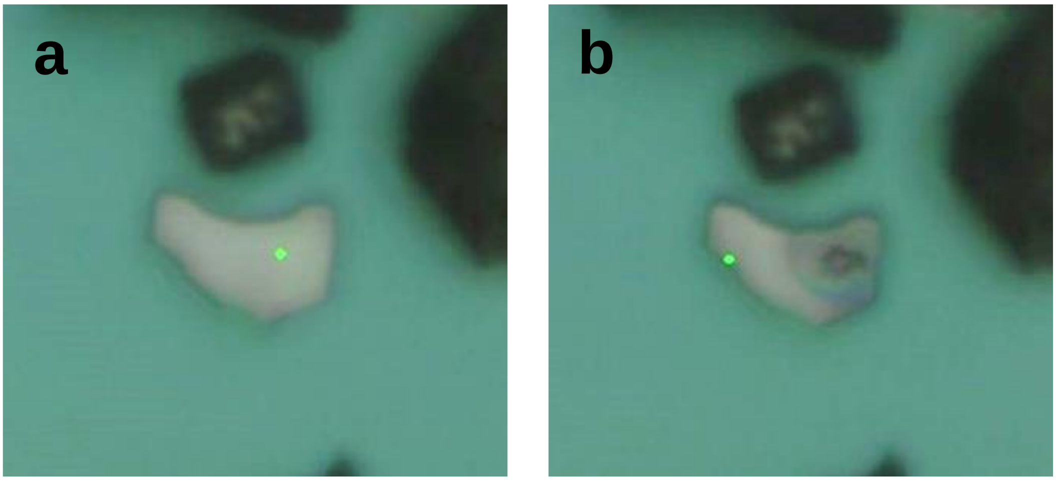

Supplementary Figure 8. Damage to VS_2 flakes during Raman measurements. Optical images of VS_2 flakes (a) before and (b) after laser illumination (~5 mW for 240 sec) when exposed to the atmosphere (green spot is the laser spot).

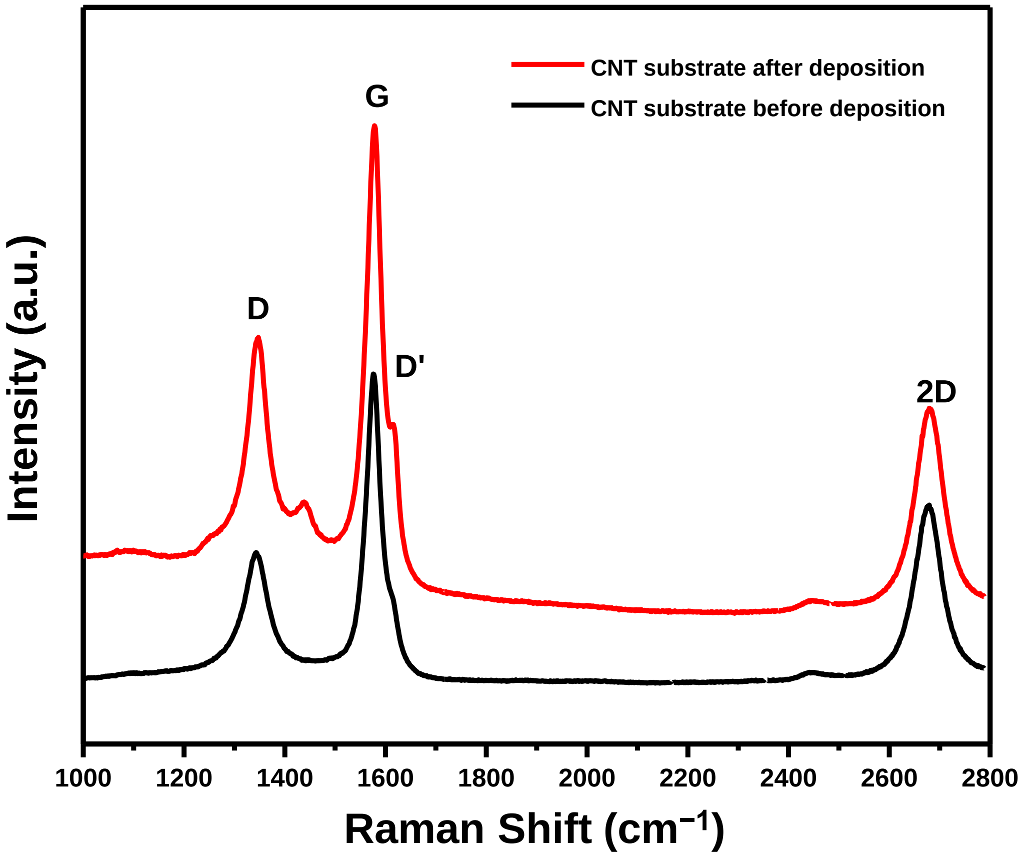

Supplementary Figure 9. Raman spectra of the CNT current collector substrate, pre and post deposition.

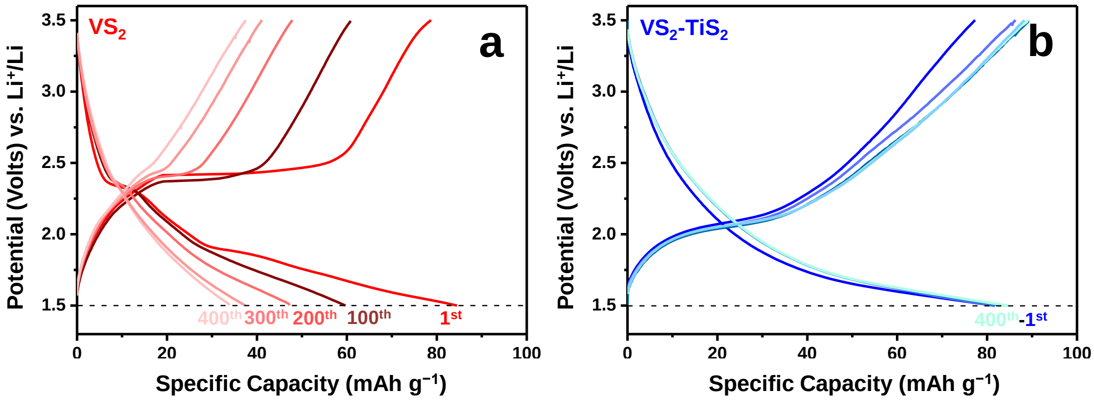

Supplementary Figure 10. Voltage profiles for the VS_2 and VS_2-TiS_2 electrodes. The charge and discharge curves for (a) VS_2 electrode and (b) VS_2-TiS_2 electrode for 1st, 100th, 200th, 300th and 400th cycles. The cells were cycled at a current density of ~1000 mA g^{-1}.

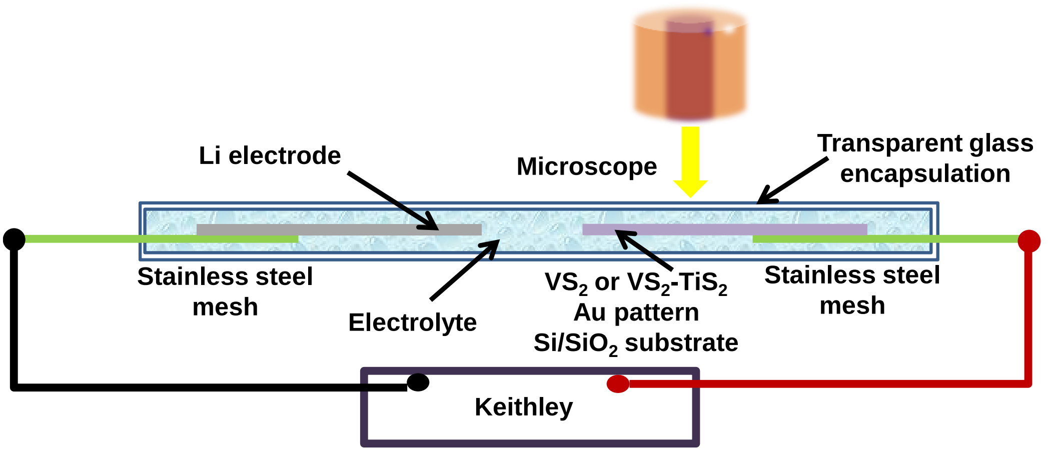

Supplementary Figure 11. Configuration of the transparent battery for *in-situ* optical observation.

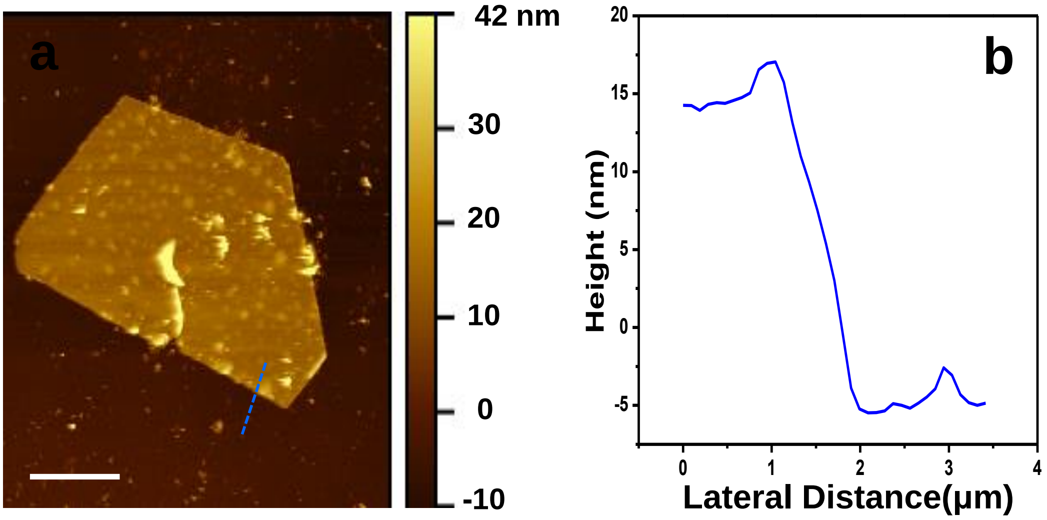

Supplementary Figure 12. AFM characterization. (a) AFM image (scale bar = 5 μm) and (b) line profile for the VS_2 platelet used for *in-situ* optical observation.

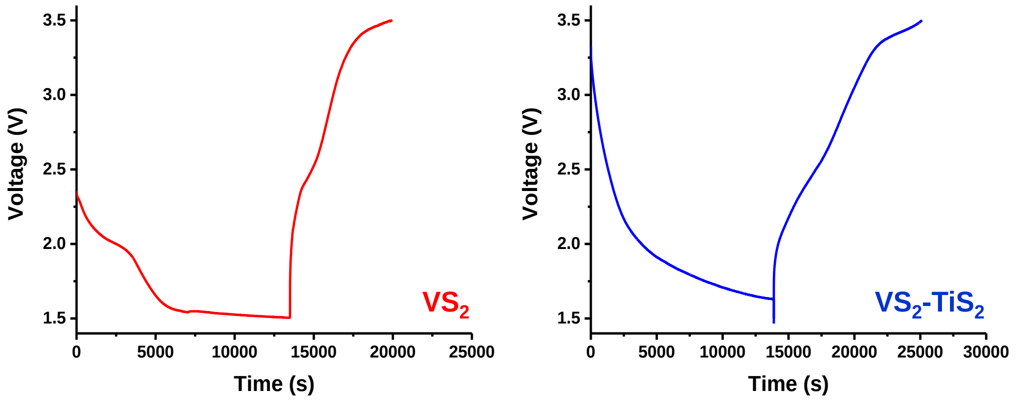

Supplementary Figure 13. Charge and discharge curves for transparent cells used for *in situ* optical characterization. Left plot is for the cell with the VS_2 electrode and the plot to the right is for the VS_2-TiS_2 electrode.

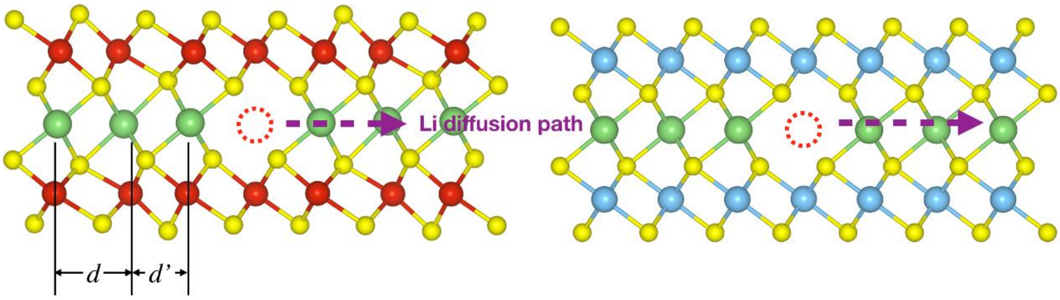

Supplementary Figure 14. DFT calculations for partially intercalated systems, which contains one Li per unit of VS_2 or TiS_2. Predicted lattice distortion for $LiVS_2$ (left) and $LiTiS_2$ (right).

Supplementary Tables

Supplementary Table 1. Raman spectroscopy data for VS_2 flakes from several references. The literature indicates that there is large scatter in the Raman data for VS_2.

Authors	Method	Raman peak positions (cm^{-1})
This work	CVD VS_2	263, 304, 379
	ALD TiS_2 on VS_2	260, 303, 324, 379
Ji *et al.*[4]	CVD VS_2	120, 168, 180, 252, 305, 330
Yuan *et al.*[5]	CVD VS_2	150, 260, 330
Liang *et al.*[6]	Hydrothermal VS_2	280, 404
Liu *et al.*[7]	Hydrothermal VS_2	140, 192, 282, 407, 688, 993
Masikhwa *et al.*[8]	Hydrothermal VS_2	138, 190, 278, 404, 686, 990
Fang *et al.*[9]	Hydrothermal VS_2	282, 302, 389, 406
Sun *et al.*[10]	Hydrothermal VS_2	282, 389, 406
Rantho *et al.*[11]	Hydrothermal VS_2	140, 285, 405

Supplementary Notes

There are three main reasons for the large scatter reported in Supplementary Table 1:

1. The first reason is the manufacturing process. Hydrothermal and chemical vapor deposition (CVD) methods have different nucleation processes. Hydrothermal method for 2D materials synthesis usually starts from small nuclei that grow into nanosheets by following the oriented attachment mechanism[10, 12, 13]. On the other hand, the CVD process is usually governed by the layer-by-layer (LBL) growth model[14, 15]. In case of VS_2 synthesis, these different growth mechanisms could introduce different stacking arrangements. Also, excess V atoms are expected to reside as intercalators between CVD grown VS_2 layers[4]. Such structural distortions can interfere with the in-plane and out-of-plane vibration, causing the peak positions to shift. Note that the Raman peaks that we report are comparable[4, 5] to other CVD grown VS_2.

2. It has been claimed in literature that the flake curvature influences the Raman response. In particular, the intense peak of the in-plane mode (282 cm^{-1}) has been attributed to the curvature of VS_2 flakes[9]. Simulation studies also indicate that the relative intensity between the E_{1g} and A_{1g} modes of 2D transition metal dichalcogenides are very sensitive to the laser set-up (polarization set-up) and could be tuned from 0 to infinity[16]. Based on the above studies, the lower intensity of the in-plane mode (~263 cm^{-1}) for our VS_2 as compared to the out-of-plane mode (~379 cm^{-1}) can be attributed to the low curvature of our VS_2 flakes as well as the polarization angle of the laser in our testing. Further in-depth experiments will be necessary to quantify the effects of structural changes and laser polarization on the relative intensities of the Raman peaks.

3. Another important reason for the large variation in Raman observation on VS_2 flakes among different published works is that VS_2 flakes are sensitive to the laser intensity and may decompose or react with oxygen if the characterization is conducted in atmosphere. This has been observed by both us (Supplementary Figure 7) and other scientists[5]. For this particular study, a vacuum optical-cryostat was used for our Raman characterization to minimize such effects.

Supplementary References

1. Mendialdua, J., Casanova, R. & Barbaux, Y. XPS studies of V_2O_5, V_6O_{13}, VO_2 and V_2O_3. *J. Electron Spectrosc. Relat. Phenom.* **71**, 249–261 (1995).
2. Yang, T., Nori, S., Zhou, H. & Narayan, J. Defect-mediated room temperature ferromagnetism in VO_2 thin films. *Appl. Phys. Lett.* **95**, 102506 (2009).
3. Lindberg, B *et al.* Molecular spectroscopy by means of ESCA II. Sulfur compounds. Correlation of electron binding energy with structure. *Physica Scripta* **1**, 286-298 (1970).
4. Ji, Q. *et al.* Metallic vanadium disulfide nanosheets as a platform material for multifunctional electrode applications. *Nano Lett.* **17**, 4908-4916 (2017).
5. Yuan, J. *et al.* Facile synthesis of single crystal vanadium disulfide nanosheets by chemical vapor deposition for efficient hydrogen evolution reaction. *Adv. Mater.* **27**, 5605-5609 (2015).
6. Liang, H. *et al.* Solution growth of vertical VS_2 nanoplate arrays for electrocatalytic hydrogen evolution. *Chem. Mater.* **28**, 5587-5591 (2016).
7. Liu, J.-Z. & Guo, P.-F. VS_2 nanosheets: A potential anode material for Li-ion batteries. *J. of Inorganic Mater.* **30**, 1339-1344 (2015).
8. Masikhwa, T. M. *et al.* Asymmetric supercapacitor based on VS_2 nanosheets and activated carbon materials. *Rsc Adv.* **6**, 38990-39000 (2016).
9. Fang, W. *et al.* Facile hydrothermal synthesis of VS_2/graphene nanocomposites with superior high-rate capability as lithium-ion battery cathodes. *ACS Appl. Mater. Interfaces* **7**, 13044−13052 (2015).
10. Sun, R. *et al.* Novel layer-by-layer stacked VS_2 nanosheets with intercalation pseudocapacitance for high-rate sodium ion charge storage. *Nano Energy* **35**, 396–404 (2017).
11. Rantho, M. N. *et al.* Asymmetric supercapacitor based on vanadium disulfide nanosheets as a cathode and carbonized iron cations adsorbed onto polyaniline as an anode. *Electrochimica Acta* **260**, 11-23 (2018).
12. Muralikrishna, S., Manjunath, K., Samrat, D., Reddy, V., Ramakrishnappa, T. & Nagarajud, D. Hydrothermal synthesis of 2D MoS_2 nanosheets for electrocatalytic hydrogen evolution reaction. *Rsc Adv.* **5**, 89389-89396 (2015).
13. Xie, J. *et al.* Defect-rich MoS_2 ultrathin nanosheets with additional active edge sites for enhanced electrocatalytic hydrogen evolution. *Adv. Mater.* **25**, 5807-5813 (2013).
14. Cai, Z., Liu, B., Zou, X. & Cheng, H.-M. Chemical vapor deposition growth and applications of two-dimensional materials and their heterostructures. *Chem. Rev.* **118**, 6091–6133 (2018).
15. Kang, K. *et al.* High-mobility three-atom-thick semiconducting films with wafer-scale homogeneity. *Nature* **520**, 656–660 (2015).
16. Liang, L. & Meunier, V. First-principles Raman spectra of MoS_2, WS_2 and their heterostructures. *Nanoscale* **6**, 5394-5401 (2014).
